# Cathepsin Oxidation Alters Alpha-Synuclein Processing

**DOI:** 10.3389/fneur.2019.00530

**Published:** 2019-05-22

**Authors:** Andrew W. Ferree

**Affiliations:** Neurology Department, Boston University Medical Center, Boston, MA, United States

**Keywords:** Parkinson's disease, alpha synuclein, lysosome, chaperone-mediated autophagy, synuclein pores, membrane permeabilization

## Introduction

The protein aggregation properties of α-synuclein (α-syn) and mitochondrial dysfunction are believed to be central to Parkinson's disease pathophysiology, however unifying mechanisms driving pathology remain uncertain. This paper proposes mitochondria-derived increased intralysosomal oxidative stress shifts cathepsin cleavage patterns to favor truncated forms of α-syn that increase its propensity to aggregate. Ultimately the shift toward intralysosomal aggregation leads to pores that destabilize lysosomal membranes and bring about cell death. The hypothesis is built on α-syn's protein structure, cathepsin cleavage profiles, aggregation properties, and processing by chaperone-mediated autophagy. This paper is not a comprehensive explanation of all α-syn biology, but rather the author's opinion on some of the most salient and important biology that's relevant to disease. In addition, this paper aims to help seed a grand unifying theory that bridges leading proposed mechanisms for Parkinson's disease (PD) pathogenesis.

## Background on α-Syn

### Structure

**Figure 1:**
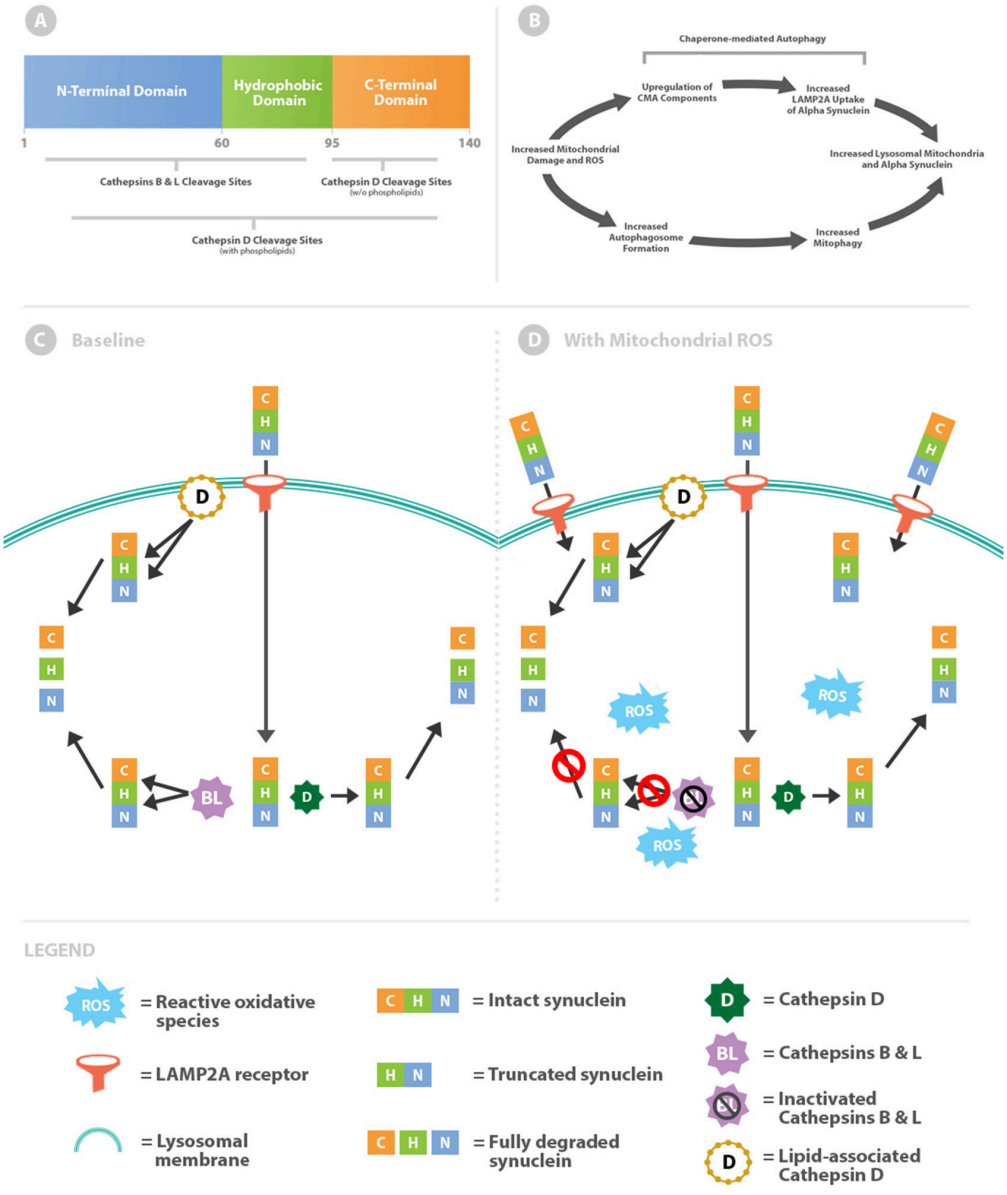
Intralysosomal oxidative stress alters cathepsin metabolism of α-syn. **(A)** Schematic of the α-syn protein highlighting differential cathepsin cleavage sites. **(B)** Mitochondrial damage simultaneously leads to increased levels of intralysosomal mitochondria and α-syn. **(C)** α-syn is imported across the lysosomal membrane via chaperone-mediated autophagy through the LAMP2A receptor. Within the lysosome, α-syn is truncated through removal of the C-terminal by cathepsin D (free solution pathway). Alternatively α-syn is more completely degraded by lipid-associated cathepsin D or cathepsins B and L. **(D)** Oxidative stress increases lysosomal import of α-syn by upregulation of chaperone-mediated autophagy. Cathepsins B and L are inactivated by reactive oxidative species, whereas cathepsin D processing (both lipid-associated and free solution) remains unaffected.

The 140 amino acids of the α-syn protein generate 3 distinct domains (Figure 1A). Residues 1–60 create an amphipathic N-terminal domain with abundant lysines and a histidine collectively resulting in a relative positive charge for this region (1). Curiously all known disease-causing point mutations cluster within the N-terminal region. Central residues 61–95 form a hydrophobic domain with affinity for membranes with increased curvature and anionic phospholipids (2–4). The final residues 96–140 create an acidic C-terminal region with several truncation sites (5, 6). Removal of negative charges with C-terminal truncation increases aggregation propensity (7) and affinity for dipolar lipids (2). As will be discussed further, this dynamic would allow α-syn to aggregate and assemble pores within membranes.

### Chaperone-Mediated Autophagy

Receptor-mediated lysosomal import of individual proteins containing selective targeting sequences, including α-syn, describes chaperone-mediated autophagy (CMA) (8). The function ascribed to CMA is to eliminate targeted proteins from the cytosol as a means of regulating expression levels. Additionally, CMA likely functions as a protective mechanism for cellular quality control (9).

Oxidative stress increases CMA activity by multiple mechanisms including upregulation of essential import components (10). Mitochondria are the major intracellular producers of reactive oxidative species (ROS) and production increases when they become damaged. Therefore, elevated levels of oxidative stress from damaged mitochondria would be expected to upregulate CMA and thereby increase α-syn within the lysosomal system. Damaged mitochondria pose a major internal threat to the cell and are also cleared via lysosomes through the process of mitophagy. Ultimately elevated lysosomal concentrations of α-syn likely coincide with increased autophagic delivery of damaged mitochondria (Figure 1B).

## Increased Intralysosomal Oxidative Stress Alters Cathepsin Cleavage of α-Syn

Delivery of damaged mitochondria would be expected to increase levels of reactive oxidative species within the lysosomal lumen. A mechanism is outlined below in which intralysosomal oxidative stress alters cathepsin-mediated processing of α-syn thereby promoting aggregation and ultimately pore formation (11, 12). A protective countermeasure is additionally outlined which may serve to prevent aggregated α-syn pores from permeabilizing the lysosomal membrane.

### Cathepsin Cleavage of α-Syn: Baseline Balance

McGlincy and colleagues identified differences in α-syn cleavage sites by cathepsins B, L, and D in free solution and in combination with phospholipids (Figure 1C) (13). In free solution, cathepsins B and L cleave throughout the length of α-syn but primarily within the N-terminus and hydrophobic regions. In contrast, cathepsin D in free solution cleaves almost exclusively in the C-terminus. By removing the negatively charged C-terminus, the truncated α-syn molecule increases oligomerization (7) and ultimately the downstream potential for pore formation.

Cathepsins B and L oppose oligomerization by virtue of cleavage sites within the N-terminal and hydrophobic regions. Targeting these regions prevents oligomerization by reducing intermolecular α-syn interactions. Moreover, cathepsin L is unique in its capacity to degrade the most stable (fibrillar) form of oligomers (13). In this baseline scenario antagonistic forces are counterbalanced, as cathepsin D promotes oligomerization and downstream pore formation whilst cathepsins B and L hinder the process (Figure 1C).

### Intralysosomal Oxidative Stress Increases α-Syn Oligomerization

Tipping the enzymatic balance toward cathepsin D activity and away from cathepsins B and L would promote α-syn aggregation and pore formation. Cathepsins B and L, but not cathepsin D, are susceptible to inactivation by oxidation from reactive carbonyls (Figure 1D) (14, 15). This effect is due to oxidation of cysteine residues within cathepsin B and L catalytic sites. Thus, a lysosomal lumen high in oxidants would shift toward cathepsin D processing of α-syn and as a consequence higher likelihood of downstream aggregation.

### Preventing Lysosomal Permeabilization by α-Syn Aggregates

The regional selectivity for cleavage between the cathepsins outlined thus far is contingent on being in free solution, which mimics the lysosomal lumen. However, cathepsin D activity is intriguingly altered when in association with membranes. In the presence of phospholipids the activity of cathepsin D broadens beyond the C-terminus to encompass the N-terminal and hydrophobic regions of α-syn (Figure 1) (13). While cathepsin D promotes oligomerization in free solution, as oligomers develop into membrane-associated pores cathepsin D can put a brake on the process. Altered activity of cathepsin D provides a critical limitation on oligomer propagation, given that baseline anti-oligomerization factors (cathepsins B and L) are inactivated by exposure to oxidized material. This regulatory mechanism is critical because pores within lysosomal membranes leads subsequent disastrous permeabilization if unchecked (16).

Advanced age is a well-known significant risk factor for PD. Moreover, all neuronal subtypes displaying α-syn pathology are unified in their abundance of lipofuscin or other oxidized lysosomal age pigments (17). It is highly probable that over time and in conditions of elevated or defective mitochondrial turnover that the capacity of lysosomal processing is exceeded and subsequent pathological cascades ensue.

## Conclusions and Future Directions

This paper proposes a simple mechanism for altered α-syn metabolism based on levels of intralysosomal oxidative stress. Mitchondria are the most significant source of ROS within cells and lysosomes are the ultimate mediators of mitochondrial degradation. The major determinant modulating the mechanistic pathway outlined here would likely be the efficiency and rate mitochondrial degradation for a given cell.

Excessive production and inability to inactivate pores could lead to disease from several pathways. Mutations or oxidative modification of target sequences could render α-syn a sub-optimal substrate for cathepsins. Impaired lysosomal capacity (18) through mutations or aging could also prevent deactivation of α-syn pores. If not inactivated, pores could promote cell death by inserting within lysosomal membranes and allowing cytoplasmic release of cathepsins. Beyond damaging individual neurons, transcellular transmission of pores is plausible as chemical blockage of lysosomal function promotes exocytosis of α-syn oligomers (19).

The two most prominent proposed mechanisms of PD pathogenesis are centered on α-syn oligomerization and mitochondrial dysfunction. Recently autophagosomal and lysosomal dysfunction have also gained traction. So far a grand unifying theory for these mechanisms has remained elusive. This paper proposes a novel mechanism connecting α-syn aggregation with mitochondrial autophagy that bridges the current theories of PD pathogensis. The multivariable mechanisms outlined here represent exciting opportunities for future discovery and drug intervention.

## Author Contributions

The author confirms being the sole contributor of this work and has approved it for publication.

### Conflict of Interest Statement

The author declares that the research was conducted in the absence of any commercial or financial relationships that could be construed as a potential conflict of interest.

## Abbreviations

α-syn: alpha synuclein
ROS: Reactive oxidative species
PD: Parkinson's disease
CMA: Chaperone mediated autophagy.
